# Performance of Hammerstein Spline Adaptive Filtering Based on Fair Cost Function for Denoising Electrocardiogram Signals

**DOI:** 10.3390/biomimetics10120828

**Published:** 2025-12-10

**Authors:** Suchada Sitjongsataporn, Theerayod Wiangtong

**Affiliations:** 1 Department of Electronic Engineering, School of Electrical and Electronic Engineering (SEE), Faculty of Engineering and Technology, Mahanakorn University of Technology, Nongchok, Bangkok 10530, Thailand; ssuchada@mut.ac.th; 2 Department of Electrical Engineering, School of Engineering, King Mongkut’s Institute of Technology Ladkrabang, Bangkok 10520, Thailand

**Keywords:** spline adaptive filtering (SAF), spline interpolation, Hammerstein function, Hammerstein spline adaptive filtering (HSAF), affine projection algorithm (APA), Fair cost function, electrocardiogram (ECG) signal

## Abstract

This paper proposes a simplified adaptive filtering approach using a Hammerstein function and the spline interpolation based on a Fair cost function for denoising electrocardiogram (ECG) signals. The use of linear filters in real-world applications has many limitations. Adaptive nonlinear filtering is a key development in tackling the challenge of discovering the specific characteristics of biomimetic systems for each person in order to eliminate unwanted signals. A biomimetic system refers to a system that mimics certain biological processes or characteristics of the human body, in this case, the individual features of a person’s cardiac signals (ECG). Here, the adaptive nonlinear filter is designed to cope with ECG variations and remove unwanted noise more effectively. The objective of this paper is to explore an individual biomedical filter based on adaptive nonlinear filtering for denoising the corrupted ECG signal. The Hammerstein spline adaptive filter (HSAF) architecture consists of two structural blocks: a nonlinear block connected to a linear one. In order to make a smooth convergence, the Fair cost function is introduced for convergence enhancement. The affine projection algorithm (APA) based on the Fair cost function is used to denoise the contaminated ECG signals, and also provides fast convergence. The MIT-BIH 12-lead database is used as the source of ECG biomedical signals contaminated by random noises modelled by Cauchy distribution. Experimental results show that the estimation error of the proposed HSAF–APA–Fair algorithm, based on the Fair cost function, can be reduced when compared with the conventional least mean square-based algorithm for denoising ECG signals.

## 1. Introduction

Electrocardiogram (ECG) signals have attracted significant interest in both medical and engineering fields [1]. ECG signals are a kind of biological signal corrupted by artefacts including patient movement and/or electrical interference. ECG signals consist of cardiac information that is essential to the diagnosis of heart diseases. With the advancement of technology, mathematical techniques can be adapted and applied to solve problems in nonlinear systems. Based on adaptive linear filtering, the finite impulse response (FIR) filter has been the conventional approach to eliminating corrupted ECG signals, including electrode motion artefacts and muscle artefacts [2].

Adaptive linear filtering techniques have been utilised, but have limitations for practical use since biomedical signals in real-world applications are generally dominated by nonlinear characteristics and nonlinear adaptive filtering. Thus, nonlinear adaptive filtering is required in order to solve the real problems of biological signals. Therefore, adaptive nonlinear filtering is a key approach to the challenge of discovering the specific characteristics of biomimetic systems for each person in order to eliminate unwanted signals. It will be shown that there are many research projects that present the application of nonlinear adaptive filtering in the field of signal processing, such as spline adaptive filtering (SAF) and Hammerstein spline adaptive filtering (HSAF) [3,4].

As stated in [3], a block-oriented model is one of the most basic nonlinear filtering techniques represented by the Hammerstein model, sometimes indicated as a nonlinear–linear model, which can be applied for biological nonlinear system identification. According to the adaptive approach, adaptive nonlinear system identification based on SAF has been achieved using a Hammerstein model comprising spline interpolation [4], which can determine the characteristic of biomedical signals in practice. As stated in the M-estimator algorithm, the adaptation of location M-estimators has also been undertaken [5,6]. As mentioned in the spline interpolation function [7], the curve of the spline basis matrix is a representation of a local interpolating approach and also uses continuous function to calculate the local parameter approximation from biomedical input signals. This approach is well-suited to capturing biological mechanisms and implementation in the field of biomedical system identification.

With assistance from the spline interpolation function and adaptive filtering, HSAF has been introduced to suppress both known and unknown disturbance sources. A common type of spline interpolation method used in many applications is the cubic spline, which can produces a smooth, natural-looking curve by applying low-degree polynomials to small data segments [8,9]. In [8], a cubic spline algorithm is used for fitting a flight path and smoothing the processing of the path planning of unmanned aerial vehicles using a bio-inspired spider–wasp algorithm. In [9], cubic spline interpolation was used for the collaborative path enhancement of multi-autonomous underwater vehicles’ collision avoidance. In [10], an improved HSAF was designed to extract the ocular noise from electroencephalogram signals in healthcare specialities. Meanwhile, HSAFs are nonlinear adaptive filters used in engineering research, such as the nonlinear system identification [11] against the impulsive noise environment [5], nonlinear acoustic echo-cancellation [12], and full-duplex communication at 2.4 GHz [13], and so on.

HSAF [4] comprises two main connected parts. The first part is nonlinear adaptive filtering, with the adaptive control point coefficients implemented in the form of a lookup table (LUT) that uses the coefficients of control point vectors defined by the spline interpolation function. The second part is adaptive linear FIR filtering with an optimisation scheme. Later research extended the design by adding a nonlinear filter structure to the original HSAF structure, which is called the Nonlinear–Linear–Nonlinear (NLN) model [3]. The experimental results show that it has a good ability to solve problems for simulation situations using real working conditions. The NLN structure is called a cascade Hammerstein spline adaptive filtering (CHSAF) structure. A previous study extended the design by adding a nonlinear filter structure to the original NLN-HSAF structure [3]. The experimental results show that it performed effectively in solving problems in simulation situations using real working conditions. The CHSAF model comprises the cascade of a memoryless nonlinear Hammerstein function, a linear Wiener filter, and a second memoryless nonlinear function, which can be considered as the cascade of a nonlinear Hammerstein system connected to a Wiener system and nonlinear system [3].

To reduce computational complexity while processing, the Fair cost function [14] has been modified for monitoring the estimated error and improving the robustness compared with the quadratic cost function in [14]. The affine projection algorithm (APA) is a stochastic algorithm that has fast convergence efficiency [15]. APA combines the advantages of normalised least mean square (NLMS) and recursive least square (RLS) algorithms, which have lower complexity and achieve faster convergence between RLS and NLMS methods in the adaptive filtering [16]. In [17], the authors improved this APA with robustness against the impulse noise. In [18], the improved APA performed with rapid convergence under non-Gaussian colour noise for sparse system identification. For fast convergence and robustness, a variable step-size technique is an important method that can support the achievement of coefficient optimisation and the inclusion of unconstrained criteria against noisy inputs and impulsive environments [19].

Therefore, HSAF applies the concepts derived from signal processing to biological signals. The goal is to apply mathematics to solve the interference present in biomedical information. The advantages of proposed HSAF-based scheme are its adaptive approach, versatile solutions for nonlinear systems, and ability to perform specific characteristic functions of biomimetic systems to defend against corrupted signals. This paper focuses on a nonlinear adaptive filter design using a HSAF structure with the help of APA based on the Fair cost function for achieving a fast mean square error convergence rate. We propose utilising this HSAF-based model, which has achieved notable success in many engineering fields, to optimise the Fair cost function for the more efficient denoising of ECG signals compared to conventional linear filtering based on a least mean square approach. The objective of this work is to explore the use of a biomedical filter based on HSAF for denoising contaminated ECG signals.

## 2. Hammerstein Spline Adaptive Filter

**Figure 1:**
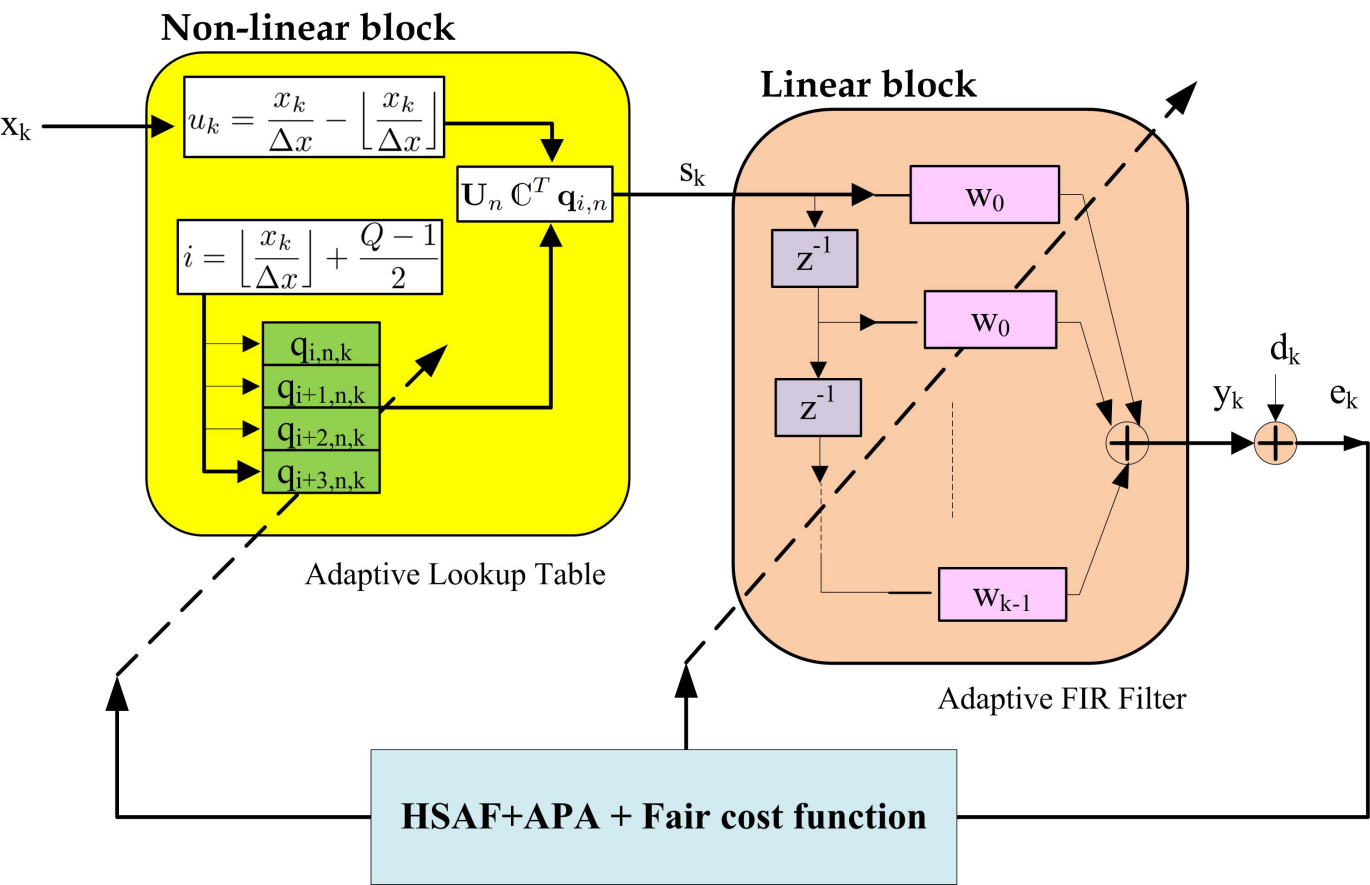
Proposed HSAF–APA–Fair architecture.

The architecture of the Hammerstein spline adaptive filter (HSAF) consists of two structural blocks: a nonlinear Hammerstein structure connected to a linear adaptive filtering, as shown in Figure 1. The nonlinear block, implemented as an adaptive lookup table (LUT), uses a spline function for interpolation with the first coefficient $q_{i,n}$. The linear structure block is an adaptive linear finite impulse response (FIR) type of linear structure for the coefficient $w_{n}$.

From Figure 1, the input signal $x_{n}$ is the input vector of the adaptive LUT block, while the output signal $s_{n}$ of the LUT block becomes the input of the linear adaptive FIR filter block

(1) $$\begin{array}{cc} s_{n} & =u_{n}^{T}\;C\;q_{i,n}\;, \end{array}$$

(2) $$\begin{array}{cc} s_{n} & ={\left[s_{n}\;\;s_{n-1}\;\;\ldots \;\;s_{n-N+1}\right]}^{T}\;, \end{array}$$

(3) $$\begin{array}{cc} i & =\left\lfloor \frac{x_{n}}{\Delta x}\right\rfloor +\frac{Q-1}{2}\;, \end{array}$$

(4) $$\begin{array}{cc} u_{n} & =\frac{x_{n}}{\Delta x}-\left\lfloor \frac{x_{n}}{\Delta x}\right\rfloor \;, \end{array}$$

(5) $$\begin{array}{cc} u_{n} & ={\left[u_{n}^{3}\;\;u_{n}^{2}\;\;u_{n}\;\;1\right]}^{T}\;, \end{array}$$

where Δx is the coefficient between two consecutive control point coefficients and *Q* is the number of coefficients used. ⌊·⌋ is the floor operator and *C* is a spline basis matrix [4], with $u_{n}$ being the coordinate between two points with the coefficient vector of the nonlinear filter.

Hence, the error $e_{n}$ at the output can be expressed by

(6) $$\begin{array}{cc} e_{n} & =d_{n}-w_{n}^{T}\;s_{n}\;, \end{array}$$

where $s_{n}$ is evaluated by the spline interpolation in (2).

Following [4], based on the minimised cost function J(q,w), we have

(7) $$\begin{array}{c} J\left(q,w\right)=\frac{1}{2}\min_{q,w}e_{n}^{2}\;, \end{array}$$

where $e_{n}$ is given in (6).

As stated in the Hammerstein function, the least mean square (LMS) method is applied on adaptive weight coefficients $q_{i,n}$

(8) $$\begin{array}{cc} q_{i,n+1} & =q_{i,n}-\mu_{q}\;\frac{\partial J(q,w)}{\partial q_{i,n}}\;, \end{array}$$

where $\frac{\partial J(q,w)}{\partial q_{i,n}}$ is given by

(9) $$\begin{array}{c} \frac{\partial J(q,w)}{\partial q_{i,n}}=\frac{1}{2}\;\frac{\partial \;{\left(d_{n}-w_{n}^{T}\;s_{n}\right)}^{2}}{\partial q_{i,n}}=-u_{n}^{T}\;C\;w_{n}\;e_{n}\;, \end{array}$$

where the Catmull–Rom (CR) spline basis matrix C that represents a local interpolating scheme [4] is

(10) $$\begin{array}{c} C=\frac{1}{2}\left[\begin{array}{cccc} -1 & 3 & -3 & 1\\ 2 & -5 & 4 & -1\\ -1 & 0 & 1 & 0\\ 0 & 2 & 0 & 0 \end{array}\right]\;. \end{array}$$

Substituting (9) into (8), we have

(11) $$\begin{array}{cc} q_{i,n+1} & =q_{i,n}+\mu_{q}\;u_{n}^{T}\;C\;w_{n}\;. \end{array}$$

In a similar fashion, the adaptive linear coefficient $w_{n}$ is defined by

(12) $$\begin{array}{cc} w_{n+1} & =w_{n}+\mu_{w}\;\frac{\partial J(q,w)}{\partial w_{n}}\;, \end{array}$$

where $\frac{\partial J(q,w)}{\partial w(k)}$ is expressed by

(13) $$\begin{array}{c} \frac{\partial J(q,w)}{\partial w_{n}}=\frac{1}{2}\;\frac{\partial \;{\left(d_{n}-w_{n}^{T}\;s_{n}\right)}^{2}}{\partial w_{n}}=-s_{n}\;e_{n}\;, \end{array}$$

By substituting (13) into (12), we arrive at

(14) $$\begin{array}{cc} ∴w_{n+1} & =w_{n}+\mu_{w}\;s_{n}\;e_{n}\;, \end{array}$$

where $\mu_{q}$ and $\mu_{w}$ denote the learning rates for $q_{i,n+1}$ and $w_{n}$, respectively.

## 3. Proposed HSAF with Affine Projection Algorithm Based on Fair Cost Function

This section introduces an adaptive nonlinear HSAF with an affine projection algorithm (APA) based on the Fair cost function (APA–Fair), which is designed to provide a specific characteristic in biomimetic systems. Initially, the Fair cost function is briefly introduced. The HSAF–APA–Fair algorithm is then developed in detail based on the HSAF model.

Following [14], the minimised Fair cost function stands on the approximate coefficient ${\tilde{w}}_{n}$ at symbol *k*, as follows:

(15) $$\begin{array}{cc} {\tilde{J}}_{n} & =\alpha^{2}\;\left\{\frac{|{\tilde{e}}_{n}|}{\alpha }-\log\left(1+\frac{|{\tilde{e}}_{n}|}{\alpha }\right)\right\}\;, \end{array}$$

where α>0 is a threshold value.

The approximate a priori error ${\tilde{e}}_{n}$ at symbol *n* is related to the desired symbol $d_{n}$

(16) $$\begin{array}{c} {\tilde{e}}_{n}=d_{n}-y_{n}=d_{n}-{\tilde{w}}_{n-1}^{T}\;s_{n}\;, \end{array}$$

where $y_{n}$ is the output signal, ${\tilde{w}}_{n}$ is the estimated weight vector, and $s_{n}$ is given in (2).

To minimise the criterion based on the Fair cost function in (15), we assume that the adaptive tap-weight vector ${\tilde{q}}_{i,n}$ and ${\tilde{w}}_{n}$ based on APA can be expressed as

(17) $$\begin{array}{cc} {\tilde{q}}_{i,n} & ={\tilde{q}}_{i,n-1}+{\tilde{\mu }}_{q_{n}}\;\frac{\nabla_{q}J_{n}}{\left(X_{n}\;X_{n}^{T}+\varrho \;I\right)}\;, \end{array}$$

(18) $$\begin{array}{cc} {\tilde{w}}_{n} & ={\tilde{w}}_{n-1}+{\tilde{\mu }}_{w_{n}}\;\frac{\nabla_{w}J_{n}}{S_{n}\;S_{n}^{T}}\;, \end{array}$$

where ${\tilde{\mu }}_{q_{n}}$ and ${\tilde{\mu }}_{w_{n}}$ are adaptive step-size for ${\tilde{q}}_{i,n}$ and ${\tilde{w}}_{n}$, respectively. $\nabla_{w}J_{n}$ and $\nabla_{q}J_{n}$ denote the gradient matrices. $X_{n}$ and $S_{n}$ are given by

(19) $$\begin{array}{cc} X_{n} & =\left[x_{n}\;\;x_{n-1}\;\;\ldots \;\;x_{n-N+1}\right]\;, \end{array}$$

(20) $$\begin{array}{cc} S_{n} & =\left[s_{n}\;\;s_{n-1}\;\;\ldots \;\;s_{n-N+1}\right]\;. \end{array}$$

Then, the gradient $\nabla_{q}{\tilde{J}}_{n}$ results from differentiating the Fair cost function in (15) with respect to ${\tilde{q}}_{i,n}$, which is given by

(21) $$\begin{array}{cc} ∴\nabla_{q}J_{n} & =\frac{\partial }{\partial {\tilde{q}}_{i,n}}\left\{\alpha^{2}\left(\frac{|{\tilde{e}}_{n}|}{\alpha }-\log\left(1+\frac{|{\tilde{e}}_{n}|}{\alpha }\right)\right)\right\}\\ \; & =\alpha^{2}\;\left(\frac{u_{n}^{T}\;C\;{\tilde{w}}_{n}}{\alpha }-\frac{u_{n}^{T}\;C\;{\tilde{w}}_{n}}{\alpha \;(1+\frac{|{\tilde{e}}_{n}|}{\alpha })}\right)=\frac{\alpha \;u_{n}^{T}\;C\;{\tilde{w}}_{n}\;{\tilde{e}}_{n}}{\alpha +|{\tilde{e}}_{n}|}\;. \end{array}$$

Moreover, the gradient $\nabla_{w}J_{n}$ is obtained by differentiating the Fair cost function in (15) with respect to ${\tilde{w}}_{n}$, which can be derived by

(22) $$\begin{array}{cc} ∴\nabla_{w}J_{n} & =\frac{\partial }{\partial {\tilde{w}}_{n}}\left\{\alpha^{2}\left(\frac{|{\tilde{e}}_{n}|}{\alpha }-\log\left(1+\frac{|{\tilde{e}}_{n}|}{\alpha }\right)\right)\right\}\\ \; & =\alpha^{2}\;\left(\frac{s_{n}}{\alpha }-\frac{s_{n}}{\alpha \;(1+\frac{|{\tilde{e}}_{n}|}{\alpha })}\right)=\frac{\alpha \;s_{n}\;{\tilde{e}}_{n}}{\alpha +|{\tilde{e}}_{n}|}\;. \end{array}$$

By replacing (21) into (17), the adaptive control points ${\tilde{q}}_{i,n}$ can be expressed as

(23) $$\begin{array}{cc} {\tilde{q}}_{i,n} & ={\tilde{q}}_{i,n-1}+\frac{{\tilde{\mu }}_{q_{n}}}{X_{n}\;X_{n}^{T}}\;\left\{\frac{\alpha \;u_{n}^{T}\;C\;{\tilde{w}}_{n}\;{\tilde{e}}_{n}}{\alpha \;+\;|{\tilde{e}}_{n}|}\right\}\;, \end{array}$$

where ${\tilde{e}}_{n}$ is given in (16). Note that the input of the nonlinear block $X_{n}$ is related to the adaptive control points ${\tilde{q}}_{i,n}$.

By substituting (22) into (18), the adaptive coefficient ${\tilde{w}}_{n}$ can be obtained by

(24) $$\begin{array}{cc} {\tilde{w}}_{n} & ={\tilde{w}}_{n-1}+\frac{{\tilde{\mu }}_{w_{n}}}{S_{n}\;S_{n}^{T}}\left\{\frac{\alpha \;s_{n}\;{\tilde{e}}_{n}}{\alpha \;+\;|{\tilde{e}}_{n}|}\right\}\;, \end{array}$$

where $s_{n}$ is given in (2). It is noticed that the input of the linear block $S_{n}$ is related to the adaptive coefficient ${\tilde{w}}_{n}$.

Following [20], adaptive step sizes ${\tilde{\mu }}_{q_{n}}$ and ${\tilde{\mu }}_{w_{n}}$ for ${\tilde{q}}_{i,n}$ and ${\tilde{w}}_{n}$ can be expressed with the help of a gradient vector, as follows:

(25) $$\begin{array}{cc} {\tilde{\mu }}_{q_{n}} & =\beta_{q}\;{\tilde{\mu }}_{q_{n-1}}+\rho_{q}\;g_{q_{n-1}}^{T}\;g_{q_{n}}\;\zeta_{n}^{2}\;, \end{array}$$

(26) $$\begin{array}{cc} {\tilde{\mu }}_{w_{n}} & =\beta_{w}\;{\tilde{\mu }}_{w_{n-1}}+\rho_{w}\;g_{w_{n-1}}^{T}\;g_{w_{n}}\;\zeta_{n}^{2}\;, \end{array}$$

(27) $$\begin{array}{cc} \zeta_{n} & =\sigma \;\zeta_{n-1}+\left(1-\sigma \right)\;{\tilde{e}}_{n}^{2}\;, \end{array}$$

where $\beta_{q}$, $\beta_{w}$, and $\rho_{q}$, $\rho_{w}$ are the smooth parameters for smooth convergence.

So, the gradient vectors $g_{q_{n}}$ and $g_{w_{n}}$, based on gradient weighted average algorithm [20], are given by

(28) $$\begin{array}{cc} g_{q_{n}} & =\psi \;g_{q_{n-1}}+\left(1-\psi \right)\;u_{n}^{T}\;C\;{\tilde{w}}_{n}\;{\tilde{e}}_{n}\;, \end{array}$$

(29) $$\begin{array}{cc} g_{w_{n}} & =\psi \;g_{w_{n-1}}+\left(1-\psi \right)\;s_{n}\;{\tilde{e}}_{n}\;, \end{array}$$

where ψ<1 and ${\tilde{e}}_{n}$ is defined in (16).

A summary of the proposed HSAF-APA based on Fair cost function (HSAF-APA-Fair) algorithm with adaptive step-size algorithm is introduced in Algorithm 1.
**Algorithm 1** Summary of proposed HSAF–APA–Fair algorithm with adaptive step-size approach
Initial value: $\tilde{w}\left(0\right)={\left[1\;0\cdots \;0\right]}^{T}$
${\tilde{q}}_{i,n}\left(0\right)=\;{\left[1\;0\cdots \;0\right]}^{T}$ and $\varrho =1\times 10^{-7}$
Fixed parameter: 0<α<1
For n=1,2,…,N
To calculate $s_{n}$ as$$\begin{array}{cc} s_{n} & =u_{n,1}^{T}\;C\;{\tilde{q}}_{i,n}\;,\\ s_{n} & ={\left[s_{n}\;\;s_{n-1}\;\;\ldots \;\;s_{n-N+1}\right]}^{T} \end{array}$$To compute $u_{n}$ and *i* as$$\begin{array}{cc} u_{n} & ={\left[u_{n}^{3}\;\;u_{n}^{2}\;\;u_{n}\;\;1\right]}^{T}\\ u_{n} & =\frac{x_{n}}{\Delta x}-\left\lfloor \frac{x_{n}}{\Delta x}\right\rfloor \\ i & =\left\lfloor \frac{x_{n}}{\Delta x}\right\rfloor +\frac{Q-1}{2} \end{array}$$To compute ${\tilde{e}}_{n}$ as$$\begin{array}{c} {\tilde{e}}_{n}=d_{n}-{\tilde{w}}_{n-1}^{T}\;s_{n} \end{array}$$To determine ${\tilde{q}}_{i,n}$ as:$$\begin{array}{cc} {\tilde{q}}_{i,n} & ={\tilde{q}}_{i,n-1}+\frac{{\tilde{\mu }}_{q_{n}}}{X_{n}\;X_{n}^{T}}\;\left\{\frac{\alpha \;u_{n}^{T}\;C\;{\tilde{w}}_{n}\;{\tilde{e}}_{n}}{\alpha \;+\;|{\tilde{e}}_{n}|}\right\}\\ X_{n} & =\left[x_{n}\;\;x_{n-1}\;\;\ldots \;\;x_{n-N+1}\right] \end{array}$$To obtain ${\tilde{\mu }}_{q_{n}}$ as$$\begin{array}{cc} {\tilde{\mu }}_{q_{n}} & =\beta_{q}\;{\tilde{\mu }}_{q_{n-1}}+\rho_{q}\;g_{q_{n-1}}^{T}\;g_{q_{n}}\;\zeta_{n}^{2}\\ g_{q_{n}} & =\psi \;g_{q_{n-1}}+\left(1-\psi \right)\;u_{n}^{T}\;C\;{\tilde{w}}_{n}\;{\tilde{e}}_{n}\\ \zeta_{n} & =\sigma \;\zeta_{n-1}+\left(1-\sigma \right)\;{\tilde{e}}_{n}^{2} \end{array}$$To calculate ${\tilde{w}}_{n}$$$\begin{array}{cc} {\tilde{w}}_{n} & ={\tilde{w}}_{n-1}+\frac{{\tilde{\mu }}_{w_{n}}}{S_{n}\;S_{n}^{T}}\left\{\frac{\alpha \;s_{n}\;{\tilde{e}}_{n}}{\alpha \;+\;|{\tilde{e}}_{n}|}\right\}\\ S_{n} & =\left[s_{n}\;\;s_{n-1}\;\;\ldots \;\;s_{n-N+1}\right] \end{array}$$To obtain ${\tilde{\mu }}_{w_{n}}$ as$$\begin{array}{cc} {\tilde{\mu }}_{w_{n}} & =\beta_{w}\;{\tilde{\mu }}_{w_{n-1}}+\rho_{w}\;g_{w_{n-1}}^{T}\;g_{w_{n}}\;\zeta_{n}^{2}\\ g_{w_{n}} & =\psi \;g_{w_{n-1}}+\left(1-\psi \right)\;s_{n}\;{\tilde{e}}_{n} \end{array}$$
end


## 4. Performance Analysis of Learning Rate

An analysis of the convergence properties of the proposed step-size parameters is conducted in virtue of a posteriori estimate error and Taylor series expansion in order to ensure the steady-state condition.

We assume a posteriori error ${\tilde{e}}_{p_{n}}$ by

(30) $$\begin{array}{c} {\tilde{e}}_{p_{n}}=d_{n}-{\tilde{w}}_{n+1}^{T}\;s_{n}={\tilde{e}}_{\min_{n}}-\delta {\tilde{w}}_{n}^{T}\;s_{n}\;. \end{array}$$

where the difference in previous and present $\delta_{{\tilde{w}}_{n}}$ is given by ${\tilde{w}}_{n}$, and can be expressed by

(31) $$\begin{array}{c} \delta {\tilde{w}}_{n}={\tilde{w}}_{n+1}-{\tilde{w}}_{n}=\frac{{\tilde{\mu }}_{w_{n}}}{S_{n}\;S_{n}^{T}}\left\{\frac{\alpha \;s_{n}\;{\tilde{e}}_{n}}{\alpha \;+\;|{\tilde{e}}_{n}|}\right\}\;. \end{array}$$

By substituting (31) into (30), a posteriori estimate error can be obtained by

(32) $$\begin{array}{c} {\tilde{e}}_{p_{n}}={\tilde{e}}_{\min_{n}}-\frac{{\tilde{\mu }}_{w_{n}}}{S_{n}\;S_{n}^{T}}\left\{\frac{\alpha \;s_{n}\;s_{n}^{T}\;{\tilde{e}}_{n}}{\alpha \;+\;|{\tilde{e}}_{n}|}\right\}\;. \end{array}$$

We assume that $|{\tilde{e}}_{p_{n}}|$≃$|{\tilde{e}}_{\min_{n}}|$ at steady state. In order to demonstrate the range of ${\tilde{\mu }}_{{\tilde{w}}_{n}}$, we take the norm of both sides of (32), which can be given by

(33) $$\begin{array}{c} ∴0<{\tilde{\mu }}_{w_{n}}<\frac{\left(S_{n}\;S_{n}^{T}\right)\cdot (\alpha +|{\tilde{e}}_{n}\left|\right)}{\alpha \;s_{n}\;s_{n}^{T}}\;. \end{array}$$

By means of Taylor series expansion, a posteriori estimate error with respect to the ${\tilde{q}}_{i,n}$ can be given by

(34) $$\begin{array}{c} {\tilde{e}\prime }_{p_{n+1}}={\tilde{e}\prime }_{p_{n}}+\frac{\partial {\tilde{e}\prime }_{p_{n}}}{\partial {\tilde{q}}_{i,n}}\cdot \delta {\tilde{q}}_{i,n}\;, \end{array}$$

where the difference in previous and present $\delta {\tilde{q}}_{i,n}$ can be expressed as

(35) $$\begin{array}{c} \delta {\tilde{q}}_{i,n}={\tilde{q}}_{i,n+1}-{\tilde{q}}_{i,n}=\frac{{\tilde{\mu }}_{q_{n}}}{X_{n}\;X_{n}^{T}}\;\left\{\frac{\alpha \;\left(u_{n}^{T}\;C\;{\tilde{w}}_{n}\right)\;{\tilde{e}}_{n}}{\alpha \;+\;|{\tilde{e}}_{n}|}\right\}\;. \end{array}$$

We demonstrate the a posteriori estimate ${\tilde{e}\prime }_{p_{n}}$ by

(36) $$\begin{array}{c} {\tilde{e}\prime }_{p_{n}}=d_{n}-{\tilde{w}}_{n+1}^{T}u_{n}C\;{\tilde{q}}_{i,n}\;, \end{array}$$

and to take the derivative on ${\tilde{e}\prime }_{p_{n}}$, with respect to ${\tilde{q}}_{i,n}$, by

(37) $$\begin{array}{c} \frac{\partial {\tilde{e}\prime }_{p_{n}}}{\partial {\tilde{q}}_{i,n}}=\frac{\partial }{\partial {\tilde{q}}_{i,n}}\left\{d_{n}-{\tilde{w}}_{n+1}^{T}u_{n}C\;{\tilde{q}}_{i,n}\right\}=-{\tilde{w}}_{n+1}^{T}\;C\;u_{n}\;. \end{array}$$

By substituting (35) and (37) into (34), the a posteriori estimate error ${\tilde{e}\prime }_{p_{n}}$ can be expressed as

(38) $$\begin{array}{c} {\tilde{e}\prime }_{p_{n+1}}={\tilde{e}\prime }_{p_{n}}-\frac{\alpha \;{\tilde{\mu }}_{q_{n}}\;\left(u_{n}^{T}\;C\;{\tilde{w}}_{n}\right)\cdot {\left(u_{n}^{T}\;C\;{\tilde{w}}_{n}\right)}^{T}\;{\tilde{e}}_{n}}{\left(X_{n}\;X_{n}^{T}\right)\cdot (\alpha +|{\tilde{e}}_{n}\left|\right)}\;. \end{array}$$

In a similar way, we obtain the range of ${\tilde{\mu }}_{q_{n}}$ by taking the norm on both sides of (38) as

(39) $$\begin{array}{c} ∴0<{\tilde{\mu }}_{q_{n}}<\frac{\left(X_{n}\;X_{n}^{T}\right)\cdot (\alpha +|{\tilde{e}}_{n}\left|\right)}{\alpha \;\left(u_{n}^{T}\;C\;{\tilde{w}}_{n}\right)\cdot {\left(u_{n}^{T}\;C\;{\tilde{w}}_{n}\right)}^{T}}\;, \end{array}$$

where we assume that all constants are positive and $|{\tilde{e}\prime }_{p_{n}}|$≃$|{\tilde{e}\prime }_{n+1}|$ at steady state.

Note that if the learning rates of ${\tilde{\mu }}_{w_{n}}$ and ${\tilde{\mu }}_{q_{n}}$ are designated as mentioned in (33) and (39), then the proposed method can be confirmed to converge at its own optimum value. These bounds in (33) and (39) are helpful to certify the convergence of the proposed approach.

## 5. Simulation Results

The efficiency of the proposed HSAF–APA–Fair algorithm was investigated using mean squared error (MSE) to assess the performance of the model and its applicability for ECG signal denoising.

### 5.1. Experimental Results of Proposed HSAF–APA–Fair Model

Random processes were simulated in the computer experiments. The performance of the proposed HSAF–APA–Fair algorithm was evaluated by comparing it to the HSAF-LMS [4] across 100 Monte Carlo trials using 7000 samples.

The input signal ($x_{n}$) is generated by

(40) $$\begin{array}{c} x_{n}=\vartheta \cdot x_{n-1}+\eta_{n}=\vartheta \cdot x_{n-1}+\sqrt{1-\vartheta^{2}}\xi_{n}\;, \end{array}$$

where $\eta_{n}$ is the random noise, $\xi_{n}$ is a zero-mean white Gaussian noise with unitary variance, and ϑ is set to [0.01, 0.99].

In the system identification, an unknown Wiener system comprising a linear component is identified as [3]:

$$\begin{array}{c} w_{0}=\left[0.6,\;-0.4,\;0.25,\;-0.15,\;0.1\right]\;. \end{array}$$

Following [5], a nonlinear memoryless target function is implemented by a 23-point length LUT $q_{0}$, which is interpolated by a uniform third-degree spline with an interval sampling Δx=0.2 as

$$\begin{array}{cc} q_{0} & =\{-2.2,-2,-1.8,\ldots ,-1.0,-0.8,-0.91,0.42,\\ & -0.01,-0.1,0.1,-0.15,0.58,1.2,1.0,1.2,\ldots ,2.0,2.2\} \end{array}$$

The initial parameters of HSAF–APA–Fair model are α=0.01; $\mu_{w}=3.75\times 10^{-2}$; $\mu_{q}=3.55\times 10^{-2}$; $\rho_{q}=2.95\times 10^{-3}$; $\rho_{w}=2.75\times 10^{-3}$; ψ=0.975; and a signal to noise ratio of SNR=25 dB. The length of the tap (*M*) is 7 for all algorithms.

The comparison of model performance between the proposed HSAF–APA–Fair method and other nonlinear models [21,22] is proved. Volterra series [21] and adaptive Hammerstein nonlinear system model by Jeraj and Mathews [22] are the representations of other nonlinear models. Volterra series [21] is a simple and powerful approach for analysing a systematic and characteristic model for a nonlinear system. Jeraj and Mathews [22] evaluated the performance of an adaptive nonlinear system by using a Hammerstein model consisting of static nonlinearity with adaptive linear filtering.

**Figure 2:**
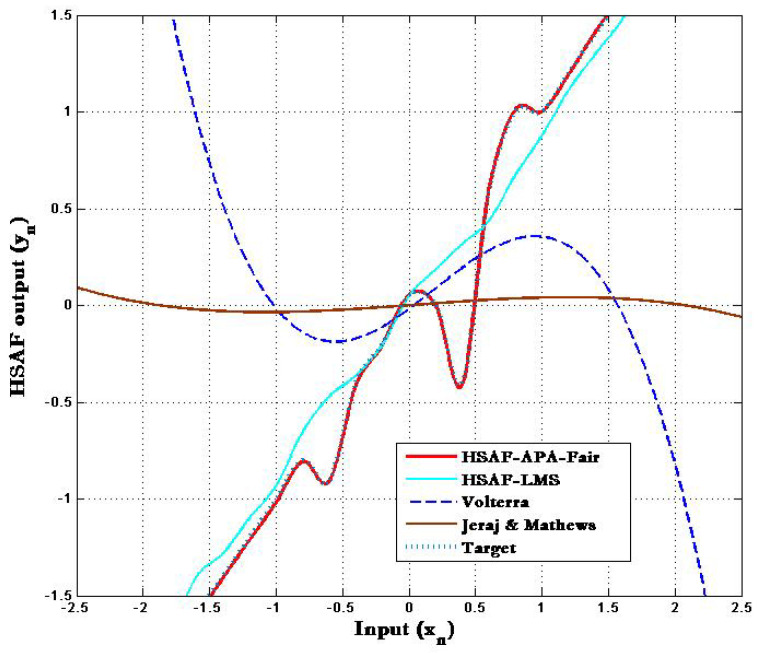
Target and adapted nonlinearities of HSAF-APA-Fair compared with HSAF-LMS [4], Volterra [21] and Jeraj & Mathews [22] with ϑ=0.15.

The adapted nonlinearity of the proposed HSAF–APA–Fair model compared with the traditional HSAF-LMS [4], Volterra [21] and Jeraj & Mathews [22] is shown in Figure 2. It can be clearly seen that the averaged results of adapted nonlinearity for Volterra [21] and Jeraj and Mathews [22] do not overlap with the nonlinear memoryless target $q_{0}$ when the traditional HSAF-LMS scheme is non-adjustable while updating at 7000 samples over 100 trials. Note that the result of the proposed HSAF–APA–Fair obtained by a spline interpolation function employed in adaptive control points ${\tilde{q}}_{i,n}$ overlies the target in order to correspond with other nonlinear algorithms.

We determine the mean square error MSE(n) that is given in dB by

(41) $$\begin{array}{c} \mathrm{MSE}\left(n\right)=10\log\left[e_{n}^{2}\right]=10\log{\left[d_{n}-w_{n-1}^{T}\;s_{n}\right]}^{2}\;. \end{array}$$

**Figure 3:**
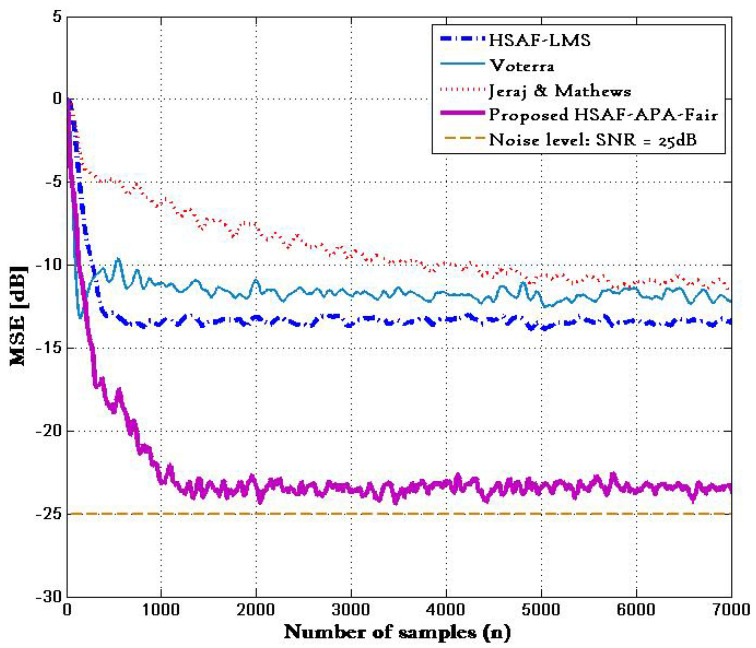
MSE curves of proposed HSAF-APA-Fair and HSAF-LMS [4], Volterra [21] and Jeraj & Mathews [22] where ϑ=0.15.

Figure 3 shows the performance of different approaches in terms of the MSE calculated by (41), where MSE curves were obtained from a test of 7000 samples with 100 trials. The comparison of MSE with ϑ=0.15 is provided and shows the MSE curves of the proposed HSAF–APA–Fair model and HSAF-LMS [4] model with ϑ=0.15 based on (40). It is clear that the MSE curves of the proposed HSAF–APA–Fair algorithm provides slower convergence compared to the traditional HSAF-LMS algorithm at steady state, while reaching the value of noise level.

**Figure 4:**
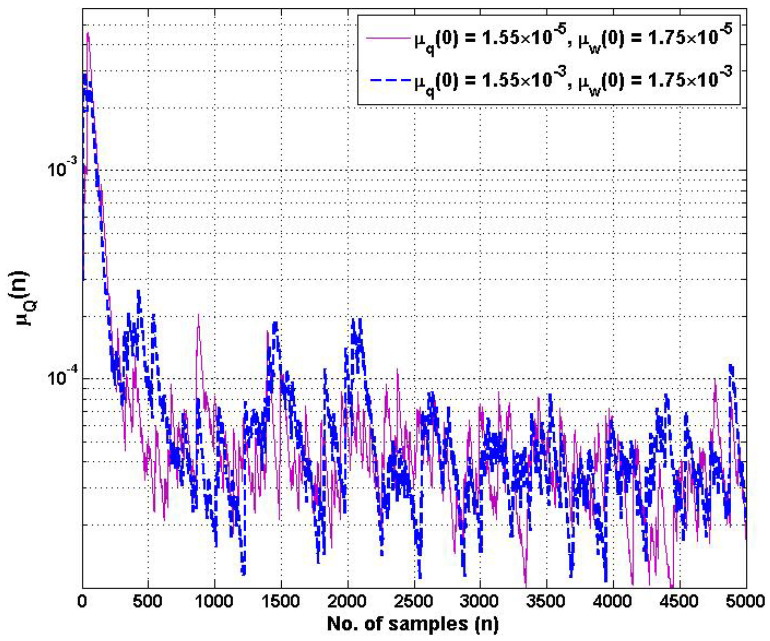
Learning rate trajectories of step-size $\mu_{q_{n}}$ of adaptive control points $q_{i,n}$ at different initial values of $\mu_{q}\left(0\right)$ and $\mu_{w}\left(0\right)$.

**Figure 5:**
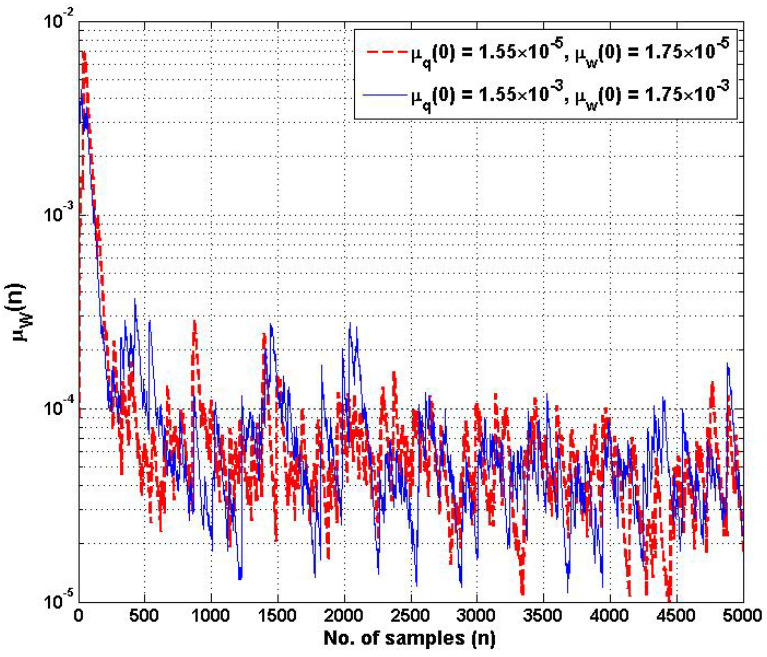
Learning rate trajectories of step-size $\mu_{w_{n}}$ of adaptive coefficient $w_{n}$ at different initial values of $\mu_{q}\left(0\right)$ and $\mu_{w}\left(0\right)$.

In addition, the learning rates of $\mu_{{\tilde{q}}_{n}}$ and $\mu_{{\tilde{w}}_{n}}$ are the crucial hyperparameters for controlling the coefficients ${\tilde{w}}_{n}$ and ${\tilde{q}}_{i,n}$ of the proposed HSAF–APA–Fair model with respect to the estimated error during updating. Figure 4 shows the learning rate of $\mu_{q_{n}}$ of adaptive control points $q_{i,n}$, with the different initial values of $\mu_{q}\left(0\right)$. Figure 5 shows the learning rate of $\mu_{w_{n}}$ of the adaptive coefficient $w_{n}$ with the different initial values of $\mu_{q}\left(0\right)$. The proposed learning rate models of step-size parameters $\mu_{q_{n}}$ and $\mu_{w_{n}}$ begin to display slight fluctuations in learning while processing after several hundreds of samples. However, both adaptive step-size models still maintain a reasonable convergence rate of MSE performance. It is noticed that the learning rate of both $\mu_{q_{n}}$ and $\mu_{w_{n}}$ can converge effectively to a steady-state environment, starting with the 1000-fold change in different initial values of each step-size.

Regarding the relationship between the MSE curves in Figure 3 and the learning rate of step-size parameters in Figure 4 and Figure 5, if the learning rate is too high at starting point, it ensures that the MSE curve of the proposed model converges quickly to find the optimal value. At steady state, we found that the learning rates adaptively were of lower values when updating the model.

### 5.2. Denoising ECG Signal

For this experiment, a dataset of ECG signals was provided by the MIT-BIH database [23,24,25]. The ECG input signal ($x_{{ECG}_{n}}$) was generated in a random process across 100 Monte Carlo trials using 7000 samples as follows:

(42) $$\begin{array}{cc} x_{{ECG}_{n}} & =\vartheta \cdot x_{{ECG}_{n-1}}+\sqrt{1-\vartheta^{2}}\xi_{n}\;. \end{array}$$

Based on statistical inference [26], the Cauchy distribution function ($\eta_{{\mathrm{Cauchy}}_{n}}$) is a representation of the random noise that appeared in the biomedical signal:

(43) $$\begin{array}{cc} \eta_{{\mathrm{Cauchy}}_{n}} & =\frac{\lambda }{\pi \cdot (x_{{ECG}_{n}}^{2}+\lambda )}\;, \end{array}$$

where λ is a scale parameter at 0.01.

Following [26], the corrupted ECG input signal ($d_{\eta_{n}}$) was contaminated by Gaussian Cauchy distribution:

(44) $$\begin{array}{c} d_{\eta_{n}}=\left(1-\lambda \right)\cdot d_{\eta_{n-1}}+\lambda \cdot \eta_{{\mathrm{Cauchy}}_{n}}\;. \end{array}$$

**Figure 6:**
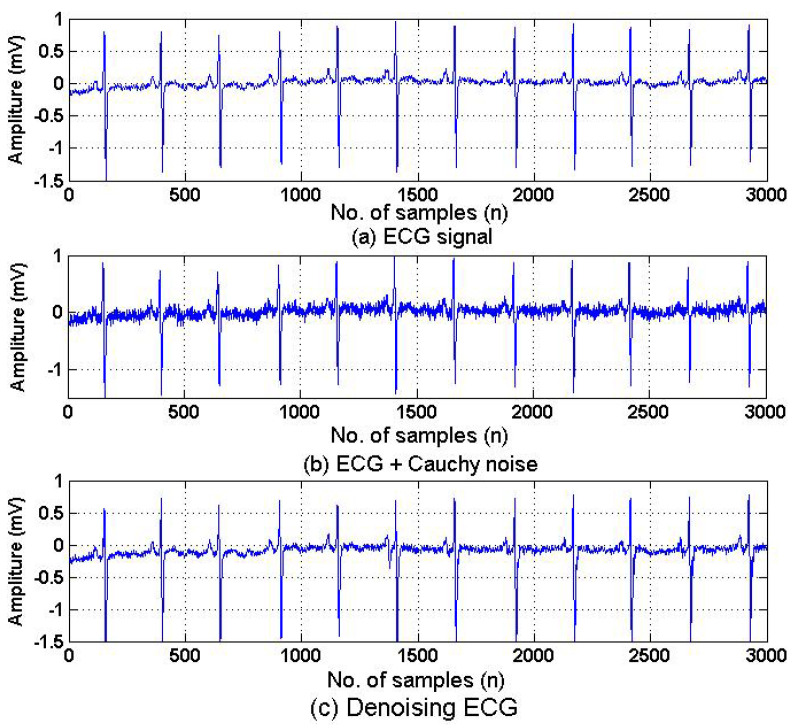
Denoised ECG signal corrupted by Gaussian Cauchy distribution function: (**a**) ECG signal; (**b**) ECG corrupted by Cauchy noise; and (**c**) denoised ECG by proposed HSAF–APA–Fair model.

Figure 6 shows the comparison between original ECG, the ECG corrupted by Gaussian Cauchy noise, and the ECG signal denoised by proposed HSAF–APA–Fair model. It can be observed that the corrupted ECG signal in Figure 6b was disturbed by Cauchy random noise. The denoised ECG signal in Figure 6c is very similar to the original ECG shown in Figure 6a after filtering using the proposed HSAF–APA–Fair method in order to eliminate the Cauchy undesired noise.

**Figure 7:**
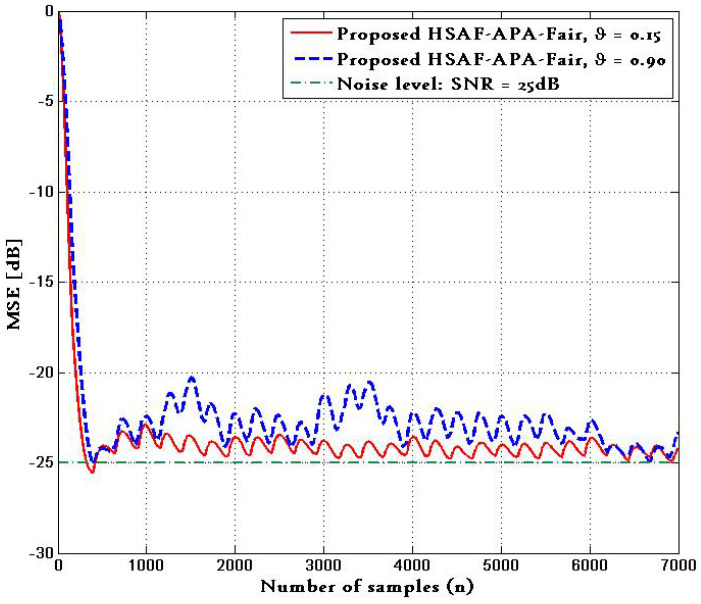
MSE curves of proposed HSAF–APA–Fair algorithm with the ECG input corrupted in Figure 6b at different ϑ=0.15,0.90.

Figure 7 shows the performance of proposed HSAF–APA–Fair algorithm with the ECG input corrupted in Figure 6b in terms of MSE, where the MSE curves were obtained from a test of 7000 samples with 100 trials. The comparison of MSE with ϑ=0.15,0.90 is provided, and shows the the MSE curves of the proposed HSAF–APA–Fair approach can converge at the steady state.

## 6. Discussion

The proposed HSAF–APA–Fair model achieves faster convergence and greater robustness to random processes compared to the traditional HSAF-LMS model. The proposed HSAF–APA–Fair model with adaptive learning rates outperforms the conventional HSAF-LMS and other nonlinear models. The shape of the adapted nonlinearity function of the proposed model can be smoothly transformed during processing. The performance of the proposed model can be optimised by step-size parameters, which were comprehensively implemented. The tuning process using an adaptive step-size algorithm aimed to enhance the convergence properties of the proposed model. This suggests that the proposed model adapts effectively after learning from a Gaussian random process. Its ability to optimise the corrupted ECG signal was tested against corruption with Cauchy noise. Despite convergence, the proposed model continues to demonstrate stable performance, capturing the underlying biomedical patterns in ECG signal and nonlinear system identification, making the proposed model a suitable option for real-world application.

## 7. Conclusions

This paper evaluates the effectiveness of the proposed HSAF–APA–Fair model, which is based on the Fair cost function for a general case of nonlinear system identification. The HSAF-based architecture incorporates a memoryless function that is adjusted during the learning process, with spline control points being automatically regulated through a gradient-based method from the APA approach. By leveraging the gradient vector for adaptive learning rate models, the step-size tuning parameters are proved. The bounds of the step-size parameters are verified with the assistance of Taylor series expansion. A systematic analysis of the learning rate of the step-size parameters was conducted to prove that the model can converge effectively to steady-state environment starting with the 1000-fold change in different initial values of each step-size parameter. Several experiments on the Gaussian random process and real data of corrupted ECG signals demonstrated the performance of the proposed HSAF–APA–Fair algorithm with regard to MSE evaluation. Simulation results show that the proposed HSAF–APA–Fair algorithm provides greater robustness and outperforms the conventional HSAF-LMS algorithm and other nonlinear systems in the tasks of system identification and ECG signal denoising.

## Data Availability

The original contributions presented in this study are included in the article. Further inquiries can be directed to the corresponding author.

## Abbreviations

The following abbreviations are used in this manuscript:

APA	Affine Projection Algorithm
ECG	Electrocardiogram
FIR	Finite Impulse Response
HSAF	Hammerstein Spline Adaptive Filtering
LMS	Least Mean Square algorithm
LUT	Lookup Table
MSE	Mean Squared Error
SAF	Spline Adaptive Filtering
